# Wealth-related inequalities in demand for family planning satisfied among married and unmarried adolescent girls and young women in sub-Saharan Africa

**DOI:** 10.1186/s12978-021-01076-0

**Published:** 2021-06-17

**Authors:** Martin K. Mutua, Yohannes D. Wado, Monica Malata, Caroline W. Kabiru, Elsie Akwara, Dessalegn Y. Melesse, Ndèye Awa Fall, Carolina V. N. Coll, Cheikh Faye, Aluisio J. D. Barros

**Affiliations:** 1 African Population and Health Research Center, Nairobi, Kenya; 2 Centre for Reproductive Health, University of Malawi, Blantyre, Malawi; 3 Institute for Global Public Health, University of Manitoba, Winnipeg, Canada; 4 International Center for Equity in Health, Federal University of Pelotas, Pelotas, RS Brazil; 5 World Health Organization, Geneva, Switzerland

**Keywords:** Demand for family planning satisfied by modern methods, Adolescent girls and young women, Wealth inequality, Sub-Saharan Africa

## Abstract

**Background:**

The use of modern contraception has increased in much of sub-Saharan Africa (SSA). However, the extent to which changes have occurred across the wealth spectrum among adolescents is not well known. We examine poor-rich gaps in demand for family planning satisfied by modern methods (DFPSm) among sexually active adolescent girls and young women (AGYW) using data from national household surveys.

**Methods:**

We used recent Demographic and Health Surveys and Multiple Indicator Cluster Surveys to describe levels of wealth-related inequalities in DFPSm among sexually active AGYW using an asset index as an indicator of wealth. Further, we used data from countries with more than one survey conducted from 2000 to assess DFPSm trends. We fitted linear models to estimate annual average rate of change (AARC) by country. We fitted random effects regression models to estimate regional AARC in DFPSm. All analysis were stratified by marital status.

**Results:**

Overall, there was significant wealth-related disparities in DFPSm in West Africa only (17.8 percentage points (pp)) among married AGYW. The disparities were significant in 5 out of 10 countries in Eastern, 2 out of 6 in Central, and 7 out of 12 in West among married AGYW and in 2 out of 6 in Central and 2 out of 9 in West Africa among unmarried AGYW. Overall, DFPSm among married AGYW increased over time in both poorest (AARC = 1.6%, p < 0.001) and richest (AARC = 1.4%, p < 0.001) households and among unmarried AGYW from poorest households (AARC = 0.8%, p = 0.045). DPFSm increased over time among married and unmarried AGYW from poorest households in Eastern (AARC = 2.4%, p < 0.001) and Southern sub-regions (AARC = 2.1%, p = 0.030) respectively. Rwanda and Liberia had the largest increases in DPFSm among married AGYW from poorest (AARC = 5.2%, p < 0.001) and richest (AARC = 5.3%, p < 0.001) households respectively. There were decreasing DFPSm trends among both married (AARC = − 1.7%, p < 0.001) and unmarried (AARC = − 4.7%, p < 0.001) AGYW from poorest households in Mozambique.

**Conclusion:**

Despite rapid improvements in DFPSm among married AGYW from the poorest households in many SSA countries there have been only modest reductions in wealth-related inequalities. Significant inequalities remain, especially among married AGYW. DFPSm stalled in most sub-regions among unmarried AGYW.

## Background

The use of modern family planning methods is effective in reducing the risk of unintended pregnancies amongst adolescent girls and young women (AGYW). AGYW aged 15–24 years have a significantly higher risk for poor health and socio-economic outcomes due to their sexual and reproductive health behavior [1, 2]. In most sub-Saharan African (SSA) countries with available data, the median age at sexual debut, first marriage, and birth is between 16 and 21 years [3, 4]. An estimated 45% of pregnancies among adolescents girls aged 15–19 in SSA are unintended [5] and result in unsafe abortions [6], school dropouts, and child marriages [7], all of which are persistent across SSA. Improving access and use of contraceptives amongst AGYW could help avert an estimated 2.1 million unintended pregnancies, 3.2 million abortions and reduce maternal mortality [5]. Additionally, it is estimated that addressing the demand for family planning satisfied with modern methods (DFPSm) for both married and unmarried adolescents aged 15–19 years could decrease fertility by 20.3% in SSA [8].

Despite a global upward trend in modern contraceptive prevalence rate among married women aged 15–49 years [9, 10], use of the methods among AGYW still remains low [11]. Data from 31 low- and middle-income countries (LMICs) from the period 2006–2014 show that unmet need for modern contraception is higher among AGYW aged 15–24 years (31%) than among women aged 25–49 (23%) [11]. Evidence further shows that unmet need for modern contraception among adolescent girls aged 15–19 years is higher than among any other age group of women [5]. Of the approximately 225 million women with an unmet need for modern contraception in LMICs, 22% are younger than 25 years [5, 12, 13] indicating that there is still a large gap in addressing the needs of adolescents and young people. Evidence also shows that differences in levels of *unmet need* among women of reproductive ages in LMICS are somewhat more pronounced *between poor* and *better*-off women, and *between* less and more educated women [11].

Concerns about the side effects of modern contraceptive methods often serve as barriers [14–16]. Furthermore, AGYW’s fear of being seen at facilities seeking family planning services and limited decision-making power within relationships also reduce their access to and use of modern contraception [16, 17]. From the supply side, lack of confidentiality on the provider’s side and poor provider attitudes are also factors limiting AGYW’s use of services [18].

The Sustainable Development Goals (SDGs) emphasize the need for leaving no one behind and ensuring universal access to sexual and reproductive health services and rights by 2030, as laid out in SDG 3 and 5 [19]. Moreover, the global Family Planning 2020 (FP 2020) [20] initiative aimed to reach an additional 120 million women and girls in the world’s poorest countries by 2020, based on the principle that all women, no matter where they live, should have access to family planning services. Evidence from Performance Monitoring and Accountability 2020 (PMA2020) data, shows that the annual rates of change in modern contraceptive prevalence among women of reproductive age (15–49 years) since 2013 varied substantially between countries from as low as 0.77 to 3.17 percentage points [21, 22]. Only six PMA settings in sub-Saharan African countries had annual growth rates higher than 1.4 percentage point change, the rate needed to achieve the FP2020 goal. Based on weighted averages, the overall absolute annual increase in modern contraceptive prevalence rates in the six settings was estimated at 1.92 percentage points for all women and 2.25 percentage points for married or cohabitating women, both higher than the FP2020 1.4 percentage point target. A few countries or subnational regions, however, had slower rates of change, less than 1.4 percentage points per year but higher than the 0.7 percentage points value at the start of FP2020 initiative.

The key question is whether and to what extent different sub-groups of AGYW from SSA benefited from the gains in contraceptive use in the last decade. Traditionally, family planning programs in SSA have focused on married adult women, with limited attention to AGYW what partly contributes to the high unmet need for contraception among unmarried, sexually active adolescents [23, 24]. Studies among women of reproductive age have shown that large inequalities exist by place of residence, level of education, socio-economic status, age, religion, and marital status [9, 10]. However, there is limited evidence regarding inequalities in contraceptive use among AGYW in SSA.

In this study, we examined levels and trends of inequalities in DFPSm among married and unmarried, sexually active AGYW in SSA countries. The primary questions are: how disadvantaged are AGYW from poorer households compared to AGYW from wealthier households in terms of DFPSm? Has there been progress in DFPSm in the past two decades; and has the progress been uniform based on wealth and marital status?

## Methods

We used data from Demographic and Health Surveys (DHS) and Multiple Indicator Cluster Surveys (MICS), which are nationally representative surveys conducted in LMICs. They both use multistage cluster sampling techniques and standardized tools for data collection [25, 26]. We used two sets of criteria to identify datasets to include in the analysis, that is, the evaluation of wealth related inequalities in DFPSm and the trend analysis. In order to evaluate the wealth-related inequalities in DFPSm, we used the most recent DHS/MICS surveys conducted since 2012. A total of 33 countries were included. Thirteen countries were excluded because they either did not have a survey during that period, or data was not publicly available. We restricted the analysis to surveys done since 2012 to get the most recent DFPSm estimates since the launch of the Family Planning 2020 (FP2020) initiative in 2012. In the second set of analyses, we included all countries with more than one survey conducted since 2000. A total of 101 surveys from 32 countries were included. Additional file 1: Table S1 in the supplementary materials, summarizes countries, and surveys included in this study. We grouped countries into four sub-regions as defined by the United Nations Population Division: Central Africa, Eastern Africa, Southern Africa, and Western Africa.

The study included women aged 15–24 years, both married women and unmarried women who reported sexual intercourse in the month preceding the interview. AGYW who reported living or cohabiting with a male partner were classified as being married while those who reported that they are divorced/separated were classified as unmarried. The sample sizes ranged from 248 in South Africa to 3448 in Malawi for married AGYW, and from 211 in Eswatini to 1092 in Sierra Leone for unmarried sexually active AGYW (see Table 1). We excluded from the analysis surveys from 9 countries that had less than 30 sexually active unmarried AGYW.

**Table 1:** Poor-rich absolute gaps in DFPSm among AGYW in sub-Saharan Africa by marital status, sub-region, and country

Sub-regions	Married					Unmarried, sexually active			
	Country	Poorest %	Richest %	N	AD (pp)	Poorest %	Richest %	N	AD (pp)
Southern	South Africa	61.4	56.8	248	− 4.6	71.7	70.3	639	− 1.4
	Zimbabwe	85.2	84.9	1029	− 0.3	*	*		
	Eswatini	80.6	84.8	209	4.2	83.7	91.9	211	8.2
	Lesotho	63.9	74.3	719	10.4	*	*		
	Namibia	58.9	72.5	331	13.6	75.2	82.3	556	7.1
Eastern	Rwanda	74.8	68.5	583	− 6.3	*	*		
	Burundi	54.2	49.1	917	− 5.1	*	*		
	Malawi	70.8	73.1	3448	2.3	38.3	45.2	413	6.9
	Comoros	18.1	29.5	471	11.4	*	*		
	Tanzania	41.7	54.8	1111	13.1	41.0	51.4	351	10.4
	Kenya	55.6	76.1	1239	**20.5**	60.4	69.0	221	8.6
	Zambia	53.0	74.9	1543	**21.9**	33.8	42.1	421	8.3
	Mozambique	33.1	55.8	757	**22.7**	50.9	73.8	260	22.9
	Uganda	35.5	60.1	1991	**24.6**	50.4	55.7	385	5.3
	Ethiopia	46.8	81.0	1288	**34.2**	*	*		
Central	Gabon	27.9	31.5	613	3.6	49.9	65.5	801	15.6
	Chad	10.3	15	1080	4.7	12.5	28.2	214	15.7
	Congo	28.4	37.2	779	8.8	40.2	39.7	778	− 0.5
	Cameroon	42.0	54.0	606	12.0	66.6	84.4	510	**17.8**
	Congo DR	9.9	24.5	1459	**14.6**	18.8	26.5	761	7.7
	Angola	3.3	44.2	1188	**40.9**	5.5	53.0	828	**47.5**
West	Ghana	40.4	35.2	457	− 5.2	41.9	36.8	388	− 5.1
	Benin	18.1	19.4	1211	1.3	26.6	28.6	546	2.0
	Niger	31.4	44.0	820	12.6	*	*		
	Gambia	11.8	25.2	486	13.4	*	*		
	Cote d’Ivoire	18.2	32.8	673	14.6	24.6	46.6	529	**22.0**
	Togo	13.7	31.1	616	**17.4**	40.5	50.5	377	10.0
	Nigeria	17.0	34.6	1471	**17.6**	20.9	31.6	690	10.7
	Guinea	23.5	44.1	594	**20.6**	42.5	64.8	281	22.3
	Sierra Leone	27.4	50.1	1222	**22.7**	52.0	70.7	1092	**18.7**
	Liberia	20.2	44.4	710	**24.2**	30.4	38.2	949	7.8
	Mali	26.0	53.0	873	**27.0**	24.7	46.8	182	22.1
	Senegal	26.6	55.8	1058	**29.2**	*	*		

Regions		Median % Poorest	Median % Richest		AD (pp)	Median % Poorest	Median % Richest		AD (pp)
Southern		63.9	74.3		10.4	75.2	76.9		1.7
Eastern		49.9	64.3		14.4	45.7	53.6		7.8
Central		19.1	34.4		15.3	29.5	46.4		16.9
West		21.9	39.6		**17.8**	30.4	46.6		16.2

Bolded estimates are statistically significant at 5% level of significance

*AD* Absolute difference, *pp* percentage point, includes only the most recent surveys from 2012; *AGYW* Adolescent Girls and Young Women aged 15–24 years; *DFPS*m Demand for family planning satisfied by modern contraceptive methods; *Poorest* The lowest tertile obtained from assets-ownership wealth-related index from principal component analysis. *Richest* highest tertile obtained from assets-ownership wealth-related index from principal component analysis. N = Weighted sample size

*Sub-category with sample size less than 30 observation excluded from analysis

The outcome of interest, the demand for family planning satisfied by modern contraceptive methods (DFPSm) is defined as the percentage of women of reproductive age who are fecund, sexually active and have their need for family planning satisfied with modern contraceptive methods [27]. The numerator is the number of women of reproductive age who are fecund, sexually active and uses a modern method for contraception and the denominator is the total number of women of reproductive age who are fecund, sexually active and in need for contraception. The following contraceptive methods were classified as modern methods: pills, condoms (male and female), intrauterine devices (IUD), sterilization (male and female), injectables, implants, patches, diaphragms, spermicidal agents (foam/jelly), lactation amenorrhea, standard days method and emergency contraception [27, 28].

Wealth-related inequalities in DFPSm were assessed by estimating DFPSm by wealth tertiles (poor, middle, and rich). The wealth tertiles were computed by generating household wealth scores from household assets and amenities for each household using principal component analysis (PCA) [29–31]. The wealth scores were then categorized into three equal groups (tertiles) to increase the sample size among the unmarried, sexually active AGYW.

### Statistical analysis

We computed the proportion of sexually active women of reproductive age in need of family planning services using a modern contraceptive method as an indicator of DFPSm at the country level by wealth tertiles, for both married and unmarried, sexually active AGYW separately. We obtained the median and interquartile range of proportions of sexually active women of reproductive age in need of family planning services using a modern contraceptive method as an indicator of DFPSm for countries at the sub-regional level. Additionally, we assessed wealth-related inequalities using absolute difference (AD) in DFPSm between the extreme levels of wealth (richest and poorest) tertiles. We assessed statistical significance of differences between sub-groups at 95% confidence level. We fitted logistic regression models of whether family planning need has been satisfied by modern contraception or not, assuming a linear change in DFPSm coverage over time for each country after pooling together all the surveys with time (year of the survey) as a covariate. We then used the average marginal effect of time to estimate the annual average rate of change (AARC) [32]. The average marginal effect is approximately equal to the regression coefficient of a linear probability model. Logistic regression has been shown to fit well for binary data [33] and has been used to estimate trends in contraceptive prevalence rates [21]. We then fitted a random-effects linear model using the estimated DFPSm proportions at different years of surveys to estimate the overall regional AARC. AARC estimates indicate the % change in DFPSm per year. The random-effects model accounts for the heterogeneity of the DFPSm rates for each country.

## Results

### Contraceptive use among AGYW by country and sub-region

**Fig. 1:**
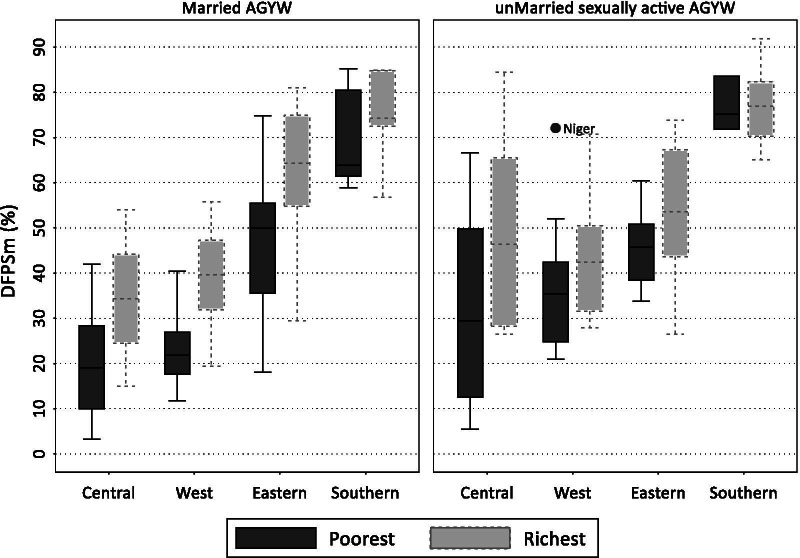
Median demand for family planning satisfied by modern methods among adolescent girls and young women aged 15–24 years in sub-Saharan Africa by marital status, sub-region, and household wealth status. *includes only the most recent surveys from 2012; *AGYW* Adolescent Girls and Young Women aged 15–24 years; *DFPSm* emand for family planning satisfied by modern contraceptive methods; *Poorest* The lowest tertile obtained from assets-ownership wealth-related index from principal component analysis. *Richest* highest tertile obtained from assets-ownership wealth-related index from principal component analysis

Demand for family planning satisfied by modern methods by wealth status for both married and unmarried, sexually active AGYW from the most recent DHS or MICS surveys are shown in Fig. 1 (sub-region means) and Additional file 2: Table S2 (country estimates and regional medians). In general, DFPSm was higher in Southern and Eastern compared to West and Central African sub-regions among both married and unmarried sexually active AGYW from poorest and richest households. Among married AGYW, DFPSm was highest in the Southern sub-region (poorest: DFPSm = 63.9% (IQR: 61.4, 80.6) and richest: DFPSm = 74.3 (72.5, 84.8)) and lowest in the Central sub-region (poorest: DFPSm = 19.1% (9.9, 28.4) and richest: DFPSm = 34.4% (24.5, 44.2). Similarly, among unmarried, sexually active AGYW, DFPSm was highest in the Southern sub-region (poorest: DFPSm = 75.2% (IQR: 71.7, 83.7) and richest: DFPSm = 76.9 (70.3, 82.3)) and lowest in West and Central sub-regions respectively. At the country level, Zimbabwe had the highest DFPSm (both poorest and richest households) while Angola and Chad had the lowest among married AGYW. Among unmarried, sexually active AGYW, Eswatini (both poorest and richest) had the highest DFPSm, while Angola, Ethiopia and Chad had the lowest DFPSm. Chad, Congo DR and Benin had the worst DFPSm among all groups marital status and wealth notwithstanding.

We observed huge disparities in DFPSm between countries. DFPSm ranged from 10% in Chad to 85% in Zimbabwe and Eswatini among married AGYM and from 21% in Chad to 86% in Zimbabwe among unmarried, sexually active AGYW. There were statistically Significant differences in DFPSm between countries with the highest overall DFPSm (Zimbabwe, Eswatini, Rwanda, Malawi, and Lesotho) and countries with the lowest overall DFPSm (Chad, Congo DR, Gambia, Benin, and Angola) among married AGYW. There were significant disparities between countries with the highest DFPSm (Eswatini, Namibia, Cameroon, and Lesotho) and countries with the lowest DFPSm (Chad, Congo DR, Rwanda, and Comoros) among unmarried, sexually active AGYW.

### Wealth-related inequalities in DFPSm

**Fig. 2:**
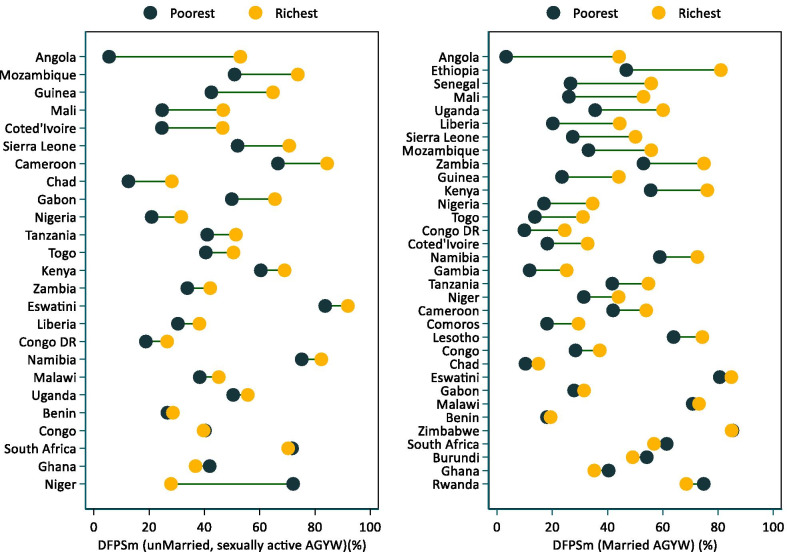
Inequality in DFPSm among AGYW in sub-Saharan Africa by country, marital status and household wealth status according to the most recent national survey. *Sorted by absolute difference between DFPSm among richest and poorest; only surveys from 2012 included; *AGYW* Adolescent Girls and Young Women aged 15–24 years; *DFPSm* Demand for family planning satisfied by modern contraceptive methods; *Poorest* The lowest tertile obtained from assets-ownership wealth-related index from principal component analysis. *Richest* highest tertile obtained from assets-ownership wealth-related index from principal component analysis. Sub-category with sample size less than 30 observation excluded from analysis

Table 1 and Fig. 2 summarize wealth-related DFPSm gaps among AGYW in SSA. There were positive differences (AGYW from richest households having higher DFPSm) in all sub-regions for both married and unmarried, sexually active AGYW with statistically significant differences in West Africa (17.8 percentage points) sub-region only among married AGYW. There were significant wealth-related disparities in DFPSm among married AGYW in five countries in the Eastern sub-region (Kenya, Zambia, Mozambique, Uganda, and Ethiopia), two countries in the Central sub-region (Congo DR and Angola), and seven countries in the West sub-region (Togo, Nigeria, Guinea, Sierra Leone, Liberia, Mali, and Senegal). Angola (40.9 percentage points), Ethiopia (34.2 percentage points), and Senegal (29.2 percentage points) had the highest poor-rich DFPSm disparities. Countries in the Southern sub-region did not present any significant poor-rich DFPSm disparities among married AGYW. South Africa, Zimbabwe, Rwanda, Burundi, and Ghana had negative, non-significant DFPSm poor-rich DFPSm disparities. There were statistically significant wealth-related DFPSm disparities among unmarried, sexually active AGYW in Angola (47.5 percentage points) and Congo DR (17.8 percentage points) in the Central sub-region and Togo (18.7 percentage points) and Nigeria (22.0 percentage points) in West sub-region. The rest of the countries with adequate sample sizes of unmarried, sexually active AGYW from both richest and poorest households had positive, non-significant poor-rich DFPSm disparities apart from South Africa (− 1.4 percentage points) and Gabon (− 0.5 percentage points). Additional file 3: Fig. S1 provides a visual representation of poor-rich DFPSm disparities.

### Trends in DFPSm

**Fig. 3:**
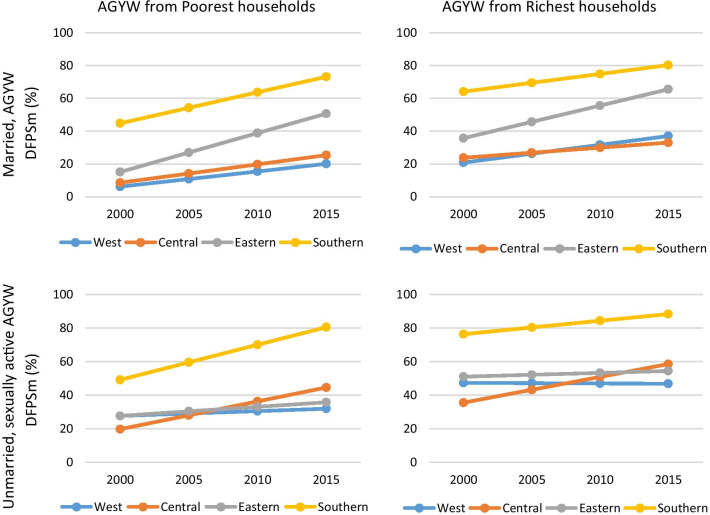
Demand for family planning satisfied among AGYW in sub-Saharan African by sub-region, marital status, household wealth status, and year. *includes all surveys from 2000; *AGYW* Adolescent Girls and Young Women aged 15–24 years; *DFPSm* Demand for family planning satisfied by modern contraceptive methods; *Poorest* The lowest tertile obtained from assets-ownership wealth-related index from principal component analysis. *Richest* highest tertile obtained from assets-ownership wealth-related index from principal component analysis

Additional file 2: Table S3 summarizes DFPSm by year of the survey for each country by marital status. Table 2 and Additional file 4: Fig. S2 (for married AGYW) and Additional file 5: Fig. S3 (for unmarried, sexually active AGYW) in the supplementary materials summarize the trends in DFPSm among the AGYW over the last two decades in the SSA countries and marital status, sub-regions, countries and household wealth status (poorest or richest) DFPSm per year. There were significant increasing trends among married AGYW from both poorest (AARC = 1.6%, p < 0.001) and richest (AARC = 1.4%, p < 0.001) households. There were significant increasing DFPSm trend among unmarried, sexually active AGYW from poorest households (AARC = 0.8%, p = 0.045). The increment among unmarried, sexually active AGYW from the richest households were not significant. At the sub-regional level, there were significant annual increasing DFPSm trends among married AGYW, both from poorest and richest households in all the four sub-regions (West, Central, Eastern, and Southern) (Table 2 and Fig. 3). Eastern sub-region had the highest annual increment among AGYW from the (poorest: AARC = 2.4%, p < 0.001, rich: AARC = 2.90%, p < 0.001). DFPSm stalled in most sub-regions among unmarried, sexually active AGYW. Southern (AARC = 2.1%, p0.030) and Central (AARC = 1.7%, p = 0.002) sub-regions had significant AARC increments among AGYW from poorest households. There was also a significant AARC increment in the Central sub-region (AARC = 1.5%, p < 0.001) among AGYW from the richest households. DFPSm stalled in the other three sub-regions among unmarried, sexually active AGYW from the richest households. There were stagnation in DFPSm among unmarried, sexually active AGYW at the country level as well as at the sub-regional level.

**Table 2:** Annual average rate of change of DFPSm among AGYW in sub-Saharan Africa by Country, marital status and household wealth status

Country	Married adolescents and young women						Unmarried adolescents and young women					
	Overall		Poorest		Richest		Overall		Poorest		Richest	
	AARC (95%CI)	p-value	AARC (95%CI)	p-value	AARC (95%CI)	p-value	AARC (95%CI)	p-value	AARC (95%CI)	p-value	AARC (95%CI)	p-value
Cameroon	1.1 (0.6;1.6)	**0.000**	2.8 (1.7;3.9)	**0.000**	− 0.2 (− 1.0;0.6)	0.619	1.9 (1.3;2.5)	**0.000**	2.5 (− 0.4;5.3)	0.088	1.5 (0.8;2.3)	**0.000**
Chad	0.5 (0.1;0.9)	**0.016**	0.7 (0.1;1.4)	**0.032**	0.3 (− 0.4;1.1)	0.346	− 0.8 (− 2.6;1.0)	0.381			0.1 (− 2.2;2.4)	0.932
Congo	1.7 (1.1;2.3)	**0.000**	1.6 (0.7;2.6)	**0.001**	1.5 (0.2;2.7)	**0.024**	1.9 (1.2;2.6)	**0.000**	1.3 (− 0.1;2.7)	0.066	1.5 (0.2;2.8)	**0.022**
Congo DR	0.9 (0.1;1.6)	**0.020**	0.6 (− 0.4;1.6)	0.240	1.0 (− 0.2;2.2)	0.091	− 0.3 (− 2.1;1.4)	0.722	− 1.0 (− 4.4;2.3)	0.542	0.0 (− 1.6;1.7)	0.962
Gabon	1.0 (0.5;1.6)	**0.000**	1.0 (0.3;1.7)	**0.004**	0.6 (− 0.4;1.6)	0.227	1.9 (1.2;2.6)	**0.000**	2.4 (1.5;3.3)	**0.000**	2.0 (0.7;3.3)	**0.003**
Burundi	2.6 (1.7;3.5)	**0.000**	3.3 (1.8;4.8)	**0.000**	2.0 (0.3;3.8)	**0.023**	*		*		*	
Ethiopia	3.3 (3.1;3.6)	**0.000**	3.0 (2.4;3.6)	**0.000**	3.5 (3.2;3.8)	**0.000**	0.6 (− 0.8;1.9)	0.429	5.3 (− 0.2;10.7)	0.057	0.6 (− 0.8;1.9)	0.429
Kenya	2.9 (2.5;3.4)	**0.000**	3.3 (2.6;4.1)	**0.000**	2.7 (2.0;3.4)	**0.000**	1.6 (0.3;2.9)	**0.013**	*		1.5 (− 0.3;3.2)	0.099
Madagascar	1.4 (− 0.1;2.9)	0.070	3.4 (0.7;6.0)	**0.014**	− 0.4 (− 2.5;1.7)	0.686	− 0.9 (− 3.0;1.2)	0.401	*		− 2.2 (− 4.7;0.3)	0.081
Malawi	2.5 (2.3;2.6)	**0.000**	2.7 (2.5;3.0)	**0.000**	2.0 (1.7;2.3)	**0.000**	1.0 (0.4;1.6)	**0.000**	0.8 (− 0.3;2.0)	0.169	0.6 (− 0.2;1.3)	0.140
Mozambique	− 0.9 (− 1.4; − 0.4)	**0.001**	− 1.7 (− 2.5; − 0.8)	**0.000**	0.0 (− 0.7;0.7)	0.972	− 0.5 (− 1.3;0.3)	0.268	− 4.7 (− 6.8; − 2.6)	**0.000**	− 0.1 (− 1.0;0.8)	0.830
Rwanda	4.6 (4.4;4.9)	**0.000**	5.2 (4.8;5.6)	**0.000**	4.0 (3.5;4.6)	**0.000**	− 2.6 (− 6.6;1.5)	0.212	*		− 2.3 (− 7.2;2.6)	0.344
Tanzania	1.3 (0.7;1.8)	**0.000**	1.7 (0.7;2.6)	**0.000**	0.5 (− 0.5;1.4)	0.357	0.7 (− 0.4;1.9)	0.220	1.2 (− 0.8;3.3)	0.244	− 0.3 (− 2.0;1.4)	0.703
Uganda	1.3 (1.0;1.6)	**0.000**	1.3 (0.8;1.8)	**0.000**	1.0 (0.5;1.5)	**0.000**	− 0.5 (− 1.1;0.2)	0.178	*		− 0.5 (− 1.3;0.3)	0.213
Zambia	1.8 (1.4;2.1)	**0.000**	2.2 (1.7;2.7)	**0.000**	1.6 (1.0;2.2)	**0.000**	− 0.4 (− 1.1;0.4)	0.339	1.0 (− 0.3;2.3)	0.127	− 0.9 (− 2.2;0.4)	0.192
Eswatini	2.3 (0.8;3.9)	**0.003**	3.2 (1.7;4.7)	**0.000**	2.7 (0.3;5.1)	**0.030**	3.0 (1.9;4.1)	**0.000**	4.4 (3.1;5.8)	**0.000**	3.1 (0.9;5.4)	**0.007**
Lesotho	2.3 (1.7;2.9)	**0.000**	3.1 (2.3;3.9)	**0.000**	1.6 (0.5;2.6)	**0.004**	1.3 (− 0.2;2.9)	0.096	*		− 0.4 (− 2.4;1.7)	0.708
Namibia	0.5 (− 0.2;1.2)	0.133	0.5 (− 0.8;1.7)	0.462	0.6 (− 0.6;1.7)	0.325	0.9 (0.3;1.5)	**0.004**	1.5 (0.3;2.7)	**0.014**	0.1 (− 0.8;0.9)	0.891
Zimbabwe	0.9 (0.5;1.3)	**0.000**	1.8 (1.1;2.4)	**0.000**	0.6 (0.1;1.1)	**0.018**	0.4 (− 1.4;2.2)	0.663	*		0.6 (− 1.4;2.6)	0.548
Benin	0.6 (0.3;0.8)	**0.000**	0.9 (0.4;1.3)	**0.000**	0.2 (− 0.2;0.6)	0.409	− 0.2 (− 0.6;0.3)	0.422	0.5 (− 0.8;1.9)	0.436	− 0.3 (− 0.9;0.3)	0.353
Burkina Faso	2.1 (1.4;2.7)	**0.000**	1.7 (0.9;2.6)	**0.000**	1.5 (0.3;2.8)	**0.019**	0.1 (− 1.7;1.8)	0.931	0.7 (− 3.0;4.5)	0.698	− 0.3 (− 2.4;1.7)	0.758
Cote d’Ivoire	0.5 (− 0.6;1.5)	0.378	− 0.2 (− 1.8;1.3)	0.768	0.7 (− 1.9;3.2)	0.604	0.7 (− 0.7;2.2)	0.319	0.0 (− 2.5;2.4)	0.996	1.5 (− 0.8;3.8)	0.196
Gambia	− 0.9 (− 3.1;1.3)	0.421	− 1.1 (− 3.6;1.4)	0.395	1.0 (− 3.0;5.1)	0.616	*		*			
Ghana	1.5 (0.9;2.2)	**0.000**	2.5 (1.5;3.5)	**0.000**	0.5 (− 0.8;1.9)	0.440	0.2 (− 0.8;1.2)	0.661	1.8 (− 1.6;5.3)	0.294	0.1 (− 1.5;1.7)	0.898
Guinea	1.2 (0.6;1.7)	**0.000**	0.7 (− 0.3;1.7)	0.157	0.7 (− 0.1;1.6)	0.094	0.8 (− 0.2;1.7)	0.113	*		0.6 (− 0.6;1.7)	0.344
Liberia	3.9 (2.5;5.4)	**0.000**	2.9 (1.2;4.5)	**0.001**	5.3 (2.9;7.7)	**0.000**	2.1 (0.9;3.3)	**0.000**	4.4 (2.0;6.8)	**0.000**	0.9 (− 0.7;2.5)	0.278
Mali	1.3 (1.0;1.5)	**0.000**	0.8 (0.3;1.2)	**0.000**	1.6 (1.2;2.0)	**0.000**	1.0 (0.4;1.6)	**0.001**	*		1.3 (0.6;1.9)	**0.000**
Niger	3.6 (2.5;4.6)	**0.000**	4.2 (2.4;6.0)	**0.000**	3.4 (1.9;4.8)	**0.000**	*		*		*	
Nigeria	0.3 (0.0;0.6)	0.094	0.3 (− 0.2;0.8)	0.198	0.1 (− 0.4;0.7)	0.648	− 1.1 (− 1.6; − 0.7)	**0.000**	− 0.7 (− 1.7;0.2)	0.133	− 1.5 (− 2.2; − 0.9)	**0.000**
Senegal	1.9 (1.6;2.3)	**0.000**	1.8 (1.3;2.3)	**0.000**	2.7 (1.8;3.5)	**0.000**	3.8 (− 2.5;10.1)	0.233	5.3 (− 0.2;10.7)	0.057	*	
Sierra Leone	2.9 (2.2;3.6)	**0.000**	2.1 (1.2;3.1)	**0.000**	3.5 (2.3;4.7)	**0.000**	2.6 (1.8;3.4)	**0.000**	− 0.8 (− 3.8;2.1)	0.572	3.1 (2.1;4.1)	**0.000**
Togo	1.5 (− 0.5;3.6)	0.145	− 1.1 (− 3.4;1.2)	0.367	0.1 (− 4.1;4.2)	0.972	0.3 (− 2.7;3.4)	0.832	5.6 (− 0.1;11.3)	0.056	0.8 (− 4.1;5.6)	0.754
West Africa	1.05 (0.56;1.54)	**0.000**	0.93 (0.52;1.33)	**0.000**	1.08 (0.31;1.85)	**0.006**	0.42 (− 0.29;1.13)	0.248	0.28 (− 0.91;1.48)	0.641	− 0.04 (− 1.18;1.11)	0.951
Central Africa	1.13 (0.77;1.48)	**0.000**	1.12 (0.03;2.22)	**0.045**	0.61 (0.26;0.97)	**0.001**	1.10 (− 0.12;2.32)	0.077	1.66 (0.63;2.68)	**0.002**	1.53 (0.94;2.13)	**0.000**
Eastern Africa	2.23 (1.14;3.31)	**0.000**	2.37 (1.23;3.51)	**0.000**	1.99 (0.90;3.07)	**0.000**	0.48 (− 0.08;1.03)	0.094	0.54 (− 0.66;1.74)	0.377	0.23 (− 0.48;0.93)	0.528
Southern Africa	1.30 (0.43;2.18)	**0.003**	1.89 (0.50;3.28)	**0.008**	1.08 (0.38;1.78)	**0.002**	1.06 (0.29;1.84)	**0.007**	2.09 (0.20;3.98)	**0.030**	0.80 (− 0.55;2.14)	0.245

95% level of significance at p < 0.05; includes all surveys from 2000; *AGYW* Adolescent Girls and Young Women aged 15–24 years; *DFPSm* Demand for family planning satisfied by modern contraceptive methods; *Poorest* The lowest tertile obtained from assets-ownership wealth-related index from principal component analysis. *Richest* highest tertile obtained from assets-ownership wealth-related index from principal component analysis; *AAARC* Annual average rate of change of DFPSm

*Sub-category with sample size less than 30 observation excluded from analysis

There were statistically significant annual increasing trend in DFPSm among married AGYW in almost all countries apart from Madagascar, Namibia, Cote d’Ivoire, Gambia, Nigeria, and Togo while the trend decreased in Mozambique. Rwanda (AARC = 4.6%), Liberia (AARC = 3.9%), Niger (AARC = 3.6%), and Ethiopia (AARC = 3.3%) had the highest AARC. Among married AGYW from the poorest households, the highest annual increment occured in Rwanda (AARC = 5.2%) and Niger (AARC = 4.2%). There was a significant negative AARC in DFPSm in Mozambique (AARC = − 1.7%, p < 0.001 points) while stagnation occured in seven countries (Namibia, Cote d’Ivoire, Gambia, Nigeria, Congo DR, Guinea, and Togo). Among married AGYW from the richest households, DFPSm stagnated in half of the countries. Liberia (AARC = 5.3%) and Rwanda (AARC = 4.0%) had the highest annual percentage point increase among married AGYW from the richest households.

Among unmarried, sexually active AGYW, a third (10 out of 29 countries) with data, had statistically significant AARC in DFPSm while two thirds (18 out of 29 countries) had no significant change. Nigeria had a statistically significant negative AARC (AARC = − 1.1%) over the period in consideration. There were statistically significant increases in AARC in DFPSm among unmarried sexually active AGYW from the poorest households in four countries (Gabon, Eswatini, Liberia, and Namibia) out of 20 countries. There were significant decreasing AARC (AARC = − 4.7%, p < 0.001) among AGYW from the poorest households in Mozambique. Only six countries (Cameroon, Congo, Gabon, Eswatini, Mali, and Sierra Leone) had significant AARC increase in DFPSm among unmarried, sexually active AGYW from the richest households, with the highest increment being in Eswatini (AARC = 3.1%, p = 0.007) and Sierra Leone (AARC = 3.1%, p < 0.001). Nigeria had a significant decreasing AARC (AARC = − 1.5%, p < 0.001).

## Discussion

This study examined wealth-related inequalities in DFPSm among AGYW using the most recent national household surveys and used surveys conducted since 2000 to examine trends in DFPSm among AGYW. The results show a general increase in DFPSm among AGYW between 2000 and 2018, but sustained existence of wealth-related inequalities in most of SSA countries more so among married AGYW. DFPSm was generally higher among unmarried, sexually active AGYW than their married counterparts. Evidence shows that unmarried sexually active young women in SSA benefited more from condom promotion programs than young married women [34]. Moreover, married AGYW are influenced by cultural norms that expect childbearing immediately following marriage to prove fertility. In almost all countries, the AGYW from the poorest households were disadvantaged in terms of DFPSm. There were statistically significant wealth-related inequalities in slightly less than half of the countries (14 out 32 countries) among married AGYW, and in 4 out 24 countries with data among unmarried, sexually active AGYW. There were statistically significant increasing trends in DFPSm among married AGYW irrespective of wealth status.

National surveys have shown an accelerated change in contraceptive use in the last two decades in most countries in SSA with increased DFPSm for all women of reproductive ages including adolescents [9, 21]. These gains are largely attributed to increased contraceptive method mix, especially the introduction of implants and increased commitments to family planning investments by the international community and national governments through initiatives such as the FP2020 [21, 35]. Our study shows that the gains of such interventions do not equally benefit AGYW. Unmarried, sexually active AGYW from the poorest households are particularly disadvantaged. These groups of AGYW face significant stigma because of their unmarried status and financial barriers that limit their access to sexual and reproductive health services [36].

The results of this study show that inequalities exist despite the overall increase in contraceptive prevalence in SSA. While sexually active unmarried AGYW had overall higher demand satisfied, trends in the last few years preceding the surveys show that the rate of increase in DFPSm is higher for currently married women. Previous studies show that unmarried, sexually active women depend on fewer methods (largely condom) than married women [37]. The recent increase in the use of long-acting reversible methods like implants [35] in SSA might, therefore, have benefited married women more, resulting in a higher increase in DFPSm among married AGYW. Further, in the few countries where there were higher increases among married AGYW from the poorest households compared to their counterparts in the richest households, there is possibility that they had programs specifically targeting the poorest women. For example, in Kenya, the Tupange program [38] focused on increasing the use of modern contraception among the poorest in urban areas and resulted in improvement in DFPSm among the poorest.

Regional variations showed that wealth-related inequalities in DFPSm were more prominent in the West and Central African (WCA) region. These results are consistent with those of previous studies that found wide disparities in DFPSm by wealth status were observed in the WCA region [9]. Studies further show that poor-rich difference in DFPS m remained the same in WCA region [10]. The substantial wealth-related inequalities by sub-regions may reflect investments by governments and other development partners in Eastern and Southern African sub-regions in promoting condom use to address the HIV/AIDS pandemic [39] thereby reaching AGYW from both the richest and poorest households. Additionally, women in WCA tend to marry earlier than women in other SSA sub-regions and might be less empowered to make decisions regarding their sexuality and reproductive health [40, 41]. Early marriages is more common among poorer AGYW than their wealthier counterparts. Moreover, AGYW from the poorest households may face greater financial barriers to accessing sexual and reproductive health services compared to richest households [42, 43].

We found a higher annual rate of increase in DFPSm among married AGYW (from both richest and poorest households) and particularly among the married AGYW from poorest households, where the magnitude of increment was higher compared to those from richest households. The improvement in DFPSm among married AGYW could be attributed to the increased attention from governments and international development partners through programs and initiatives like the FP2020 [21], which revitalized family planning in countries lagging in terms of contraceptive uptake. These initiatives and programs might have benefited married women more as their contraceptive preferences are more diverse than unmarried, sexually active AGYW [44, 45]. Unmarried, sexually active AGYW also face significant barriers in accessing family planning services [40, 46–48]. There is a need for governments and development partners to refocus their efforts and target unmarried, sexually active AGYW who are being left behind in order to improve DFPSm and achieve FP2020 and SDG targets.

Our analysis shows that DFPSm stalled in a quarter of countries among married AGYW and in a third of countries for unmarried, sexually active AGYW. Studies using PMA2020 surveys have also shown a stall in contraceptive use among unmarried, women in the same settings [21]. The stall in DFPSm suggests that a significant number of countries will not reach FP2020 targets [49] and that adequate attention is not paid to addressing inequalities. While it is important to examine why DFPSm stalled in a quarter of countries in SSA and why some groups have more demand satisfied than others, evidence from DHS and other national surveys show that only a minority of women report lack of access as a reason for unmet need. Major reasons for non-use relate to fear of side effects and health concerns, which vary by socio-economic and marital status [50]. For unmarried sexually active AGYW, side effects and health concerns are the second major reason for not using contraception after infrequent sexual activity [11]. Collectively, these findings suggest the need to prioritize unmarried, young women in programs, and to better understand their contraceptive preferences.

### Limitations

The findings of this study should be interpreted with certain limitations in mind. First, our results give a limited picture of demand for family planning satisfied by modern methods in some regions given few countries are represented and may not be generalizable. Second, the timing of surveys varied by countries so the trend analyses are over different time points. Third, the sample size of unmarried sexually active AGYW was relatively small, which may lead to large sampling errors. We did not estimate DFPSm for surveys where the sample size was less than 30 per category. For some countries, we did not estimate the DFPSm and were therefore unable to compare adolescents and young women. Despite these limitations, this study uses nationally-representative data that provide important insights into how AGYW contraceptive behavior varies by household wealth status, marital status and stratifiers within and across countries in SSA.

## Conclusions

Improvements in DFPSm among married AGYW from the poorest households in many SSA countries between 2010 and 202 have contributed to modest reductions of wealth-related inequalities in DFPSm. However, sustained efforts are needed to ensure that family planning programs reach both married and unmarried, sexually active AGYW. Further, governments and development partners need to increase investments in family programs in countries in the WCA region, which lags DFPSm among AGYW. Moreover, there is a need for programs that specifically target poor married women and unmarried sexually active AGYW to reduce inequalities in DFPSm in SSA and improve DFPSm among unmarried, sexually active AGYW which has stalled in most sub-regions.

## Supplementary Information

**Additional file 1: FigureS1. **Absolute difference in DFPSm between AGYW from richest and poorest households.

**Additional file 2: Figure S2. **Annual average rate of change of DFPSm among married AGYW (overall, poorest andrichest) by country.

**Additional file 3: Figure S3. **Annual average rate of change of DFPSm among unmarried sexually active AGYW (overall, poorest andrichest) by country.

**Additional file 4: Table S1.** Data sources and and sample sizes by year of survey and marital status.

**Additional file 5: Table S2. **DFPSm among AGYW in SSAby marital status, sub-region, country, and household wealth status.

## Data Availability

The dataset used for the current study is available for free from https://dhsprogram.com/data/available-datasets.cfm.

## Abbreviations

AARC: Annual Average Rate of Change
AD: Absolute difference
AGYW: Adolescents Girls and Young Women
DHS: Demographic and Health Surveys
ESA: Eastern and Southern Africa
DFPSm: Demand for family planning satisfied by modern contraceptive methods
IUD: Intrauterine devices
LMICs: Low and Middle-Income Countries
MICS: Multiple Indicator Cluster Surveys
PCA: Principal Component Analysis
PMA2020: Performance and Monitoring and Accountability 2020
SDGs: Sustainable Development Goals
SSA: Sub-Saharan Africa
WCA: West and Central Africa
