# Supplementary material for: Wealth-related inequalities in demand for family planning satisfied among married and unmarried adolescent girls and young women in sub-Saharan Africa
Source: Reprod Health. 2021 Jun 17;18(Suppl 1):116. doi: 10.1186/s12978-021-01076-0 (PMC8210345; doi:10.1186/s12978-021-01076-0)
Supplement: Supplementary file 2 — Additional file 2: Figure S2. Annual average rate of change of DFPSm among married AGYW (overall, poorest andrichest) by country. [file 12978_2021_1076_MOESM2_ESM.docx]

Table S2: DFPSm among AGYW in SSA by marital status, sub-region, country, and household wealth status.

|  | Married | | | | Unmarried, sexually active | | | |
| --- | --- | --- | --- | --- | --- | --- | --- | --- |
| Country | Poorest % (95% CI) | Richest % (95% CI) | Overall % (95% CI) | N | Poorest % (95% CI) | Richest % (95% CI) | Overall % (95% CI) | N |
| South Africa | 61.4 [48.4,72.9] | 56.8 [34.4,76.8] | 64.4 [55.6,72.4] | 248 | 71.7 [64.5,77.9] | 70.3 [60.1,78.7] | 68.9 [64.2,73.2] | 639 |
| Zimbabwe | 85.2 [80.5,89.0] | 84.9 [78.5,89.7] | 83.7 [80.7,86.4] | 1029 |  | 76.9 [62.6,86.8] | 68.3 [59.6,75.8] | 101 |
| Eswatini | 80.6 [71.4,87.3] | 84.8 [68.2,93.5] | 77.9 [65.5,86.7] | 209 | 83.7 [73.8,90.3] | 91.9 [79.0,97.1] | 85.9 [79.4,90.6] | 211 |
| Lesotho | 63.9 [57.2,70.1] | 74.3 [66.1,81.1] | 68.4 [64.2,72.4] | 719 |  | 65.1 [49.8,77.8] | 69.8 [58.6,79.1] | 177 |
| Namibia | 58.9 [48.8,68.4] | 72.5 [59.9,82.4] | 66.1 [60.2,71.6] | 331 | 75.2 [64.2,83.7] | 82.3 [75.6,87.4] | 79.9 [75.2,83.9] | 556 |
| Rwanda | 74.8 [68.6,80.1] | 68.5 [59.0,76.7] | 72.0 [67.7,75.9] | 583 |  | 26.5 [15.7,41.1] | 27.4 [20.1,36.1] | 110 |
| Burundi | 54.2 [47.8,60.5] | 49.1 [41.8,56.5] | 49.4 [45.5,53.3] | 917 |  |  | 50.3 [37.1,63.3] | 59 |
| Malawi | 70.8 [68.0,73.4] | 73.1 [68.8,77.0] | 71.5 [69.4,73.5] | 3448 | 38.3 [27.3,50.7] | 45.2 [36.9,53.7] | 43.8 [38.0,49.8] | 413 |
| Comoros | 18.1 [11.6,27.3] | 29.5 [21.7,38.7] | 22.2 [18.0,27.0] | 471 |  |  | 35.7 [12.4,68.5] | 48 |
| Tanzania | 41.7 [35.3,48.4] | 54.8 [47.9,61.6] | 47.7 [43.9,51.6] | 1111 | 41.0 [27.0,56.6] | 51.4 [41.8,60.9] | 51.9 [45.3,58.5] | 351 |
| Kenya | 55.6 [49.7,61.4] | 76.1 [69.2,81.8] | 66.5 [63.0,69.9] | 1239 | 60.4 [47.0,72.5] | 69.0 [53.6,81.1] | 63.4 [53.9,71.9] | 221 |
| Zambia | 53.0 [48.2,57.8] | 74.9 [69.0,79.9] | 62.4 [59.2,65.5] | 1543 | 33.8 [24.5,44.7] | 42.1 [33.0,51.8] | 34.7 [29.5,40.4] | 421 |
| Mozambique | 33.1 [24.5,42.9] | 55.8 [50.3,61.1] | 44.6 [39.8,49.5] | 757 | 50.9 [28.6,72.9] | 73.8 [67.8,79.1] | 65.4 [59.1,71.1] | 260 |
| Uganda | 35.5 [31.8,39.5] | 60.1 [54.8,65.1] | 46.9 [44.1,49.8] | 1991 | 50.4 [38.2,62.6] | 55.7 [47.3,63.9] | 53.6 [47.8,59.2] | 385 |
| Ethiopia | 46.8 [38.3,55.5] | 81.0 [75.5,85.6] | 65.6 [60.4,70.4] | 1288 |  | 65.6 [50.7,77.9] | 56.4 [42.7,69.1] | 80 |
| Gabon | 27.9 [21.7,35.1] | 31.5 [22.5,42.0] | 33.3 [27.9,39.1] | 613 | 49.9 [42.1,57.7] | 65.5 [48.4,79.4] | 57.3 [49.7,64.4] | 801 |
| Chad | 10.3 [6.9,15.2] | 15.0 [11.0,20.3] | 10.8 [8.6,13.5] | 1080 | 12.5 [4.1,32.4] | 28.2 [19.9,38.3] | 20.8 [15.3,27.6] | 214 |
| Congo | 28.4 [23.2,34.3] | 37.2 [26.6,49.3] | 33.0 [29.2,37.1] | 779 | 40.2 [32.9,48.0] | 39.7 [31.1,48.9] | 41.7 [37.4,46.2] | 778 |
| Cameroon | 42.0 [34.6,49.8] | 54.0 [44.9,63.0] | 49.1 [44.7,53.5] | 606 | 66.6 [51.9,78.6] | 84.4 [78.6,88.8] | 77.8 [73.6,81.5] | 510 |
| Congo DR | 9.9 [6.7,14.5] | 24.5 [19.9,29.7] | 15.8 [13.1,19.0] | 1459 | 18.8 [12.1,28.1] | 26.5 [21.4,32.2] | 24.2 [19.7,29.4] | 761 |
| Angola | 3.3 [1.4,7.7] | 44.2 [34.6,54.3] | 21.6 [17.8,25.8] | 1188 | 5.5 [2.8,10.6] | 53.0 [44.5,61.3] | 36.3 [31.1,41.9] | 828 |
| Ghana | 40.4 [32.0,49.3] | 35.2 [23.9,48.6] | 36.6 [30.8,42.8] | 457 | 41.9 [30.5,54.1] | 36.8 [24.7,50.9] | 36.1 [29.8,42.9] | 388 |
| Benin | 18.1 [14.0,23.2] | 19.4 [15.5,24.0] | 18.1 [15.8,20.6] | 1211 | 26.6 [19.0,35.8] | 28.6 [22.8,35.1] | 28.2 [24.4,32.3] | 546 |
| Niger | 31.4 [23.7,40.2] | 44.0 [38.2,50.0] | 35.9 [31.8,40.2] | 820 |  |  |  |  |
| Gambia | 11.8 [7.6,17.9] | 25.2 [17.5,35.0] | 17.1 [12.7,22.7] | 486 |  |  |  |  |
| Cote d’Ivoire | 18.2 [13.4,24.3] | 32.8 [23.5,43.7] | 24.5 [20.9,28.6] | 673 | 24.6 [16.1,35.6] | 46.6 [37.7,55.8] | 37.4 [31.8,43.4] | 529 |
| Togo | 13.7 [9.8,18.7] | 31.1 [24.4,38.8] | 24.8 [21.2,28.9] | 616 | 40.5 [29.5,52.6] | 50.5 [41.7,59.3] | 44.3 [38.7,50.1] | 377 |
| Nigeria | 17.0 [13.4,21.3] | 34.6 [29.2,40.5] | 27.4 [24.6,30.3] | 1471 | 20.9 [13.2,31.4] | 31.6 [26.1,37.8] | 28.4 [24.9,32.2] | 690 |
| Guinea | 23.5 [15.9,33.4] | 44.1 [35.5,53.0] | 35.2 [30.1,40.6] | 594 | 42.5 [29.4,56.7] | 64.8 [54.4,74.0] | 56.8 [49.6,63.8] | 281 |
| Sierra Leone | 27.4 [23.1,32.3] | 50.1 [43.3,56.8] | 38.0 [34.5,41.5] | 1222 | 52.0 [44.6,59.4] | 70.7 [64.3,76.3] | 65.0 [61.0,68.8] | 1092 |
| Liberia | 20.2 [15.9,25.3] | 44.4 [31.6,58.1] | 32.9 [26.2,40.3] | 710 | 30.4 [22.4,39.7] | 38.2 [30.4,46.6] | 38.4 [33.4,43.8] | 949 |
| Mali | 26.0 [19.1,34.3] | 53.0 [46.2,59.6] | 38.8 [34.7,43.1] | 873 | 24.7 [10.8,46.8] | 46.8 [36.1,57.9] | 40.0 [31.7,48.9] | 182 |
| Senegal | 26.6 [22.8,30.8] | 55.8 [47.0,64.3] | 37.1 [33.8,40.4] | 1058 |  |  | 42.3 [24.8,62.0] | 50 |
| Sub region | Median (IQR) | Median (IQR) | Median (IQR) | N | Median (IQR) | Median (IQR) | Median (IQR) |  |
| Southern | 63.9 [61.4,80.6] | 74.3 [72.5,84.8] | 68.4 [66.1,77.9] |  | 75.2 [71.7,83.7] | 76.9 [70.3,82.3] | 69.8 [68.9,79.9] |  |
| Eastern | 49.9 [35.5,55.6] | 64.3 [54.8,74.9] | 55.9 [46.9,66.5] |  | 45.7 [38.3,50.9] | 53.6 [43.7,67.3] | 51.1 [35.7,56.4] |  |
| Central | 19.1 [9.9,28.4] | 34.4 [24.5,44.2] | 27.3 [15.8,33.3] |  | 30.4 [24.7,41.9] | 46.6 [36.8,50.5] | 39.2 [36.1,44.3] |  |
| West | 21.9 [17.6,27.0] | 39.6 [32.0,47.3] | 34.1 [24.7,36.9] |  | 29.5 [12.5,49.9] | 46.4 [28.2,65.5] | 39.0 [24.2,57.3] |  |

Based on the most recent surveys from 2012; AGYW – Adolescent Girls and Young Women aged 15-24 years; DFPSm – Demand for family planning satisfied by modern contraceptive methods; Poorest – The lowest tertile obtained from assets-ownership wealth-related index from principal component analysis. Richest – highest tertile obtained from assets-ownership wealth-related index from principal component analysis. Sub-category with sample size less than 30 observation excluded from analysis.

Table S3: AGYW DFPSm by country, surveys and marital status for all countries with at least two surveys

| country | Survey Year | unMarried sexually active | | | Married | | |
| --- | --- | --- | --- | --- | --- | --- | --- |
|  |  | All | Poorest | Richest | All | Poorest | Richest |
| Benin | 2001 | 21.6 |  | 21.9 | 13.9 | 10.5 | 21.4 |
| Benin | 2006 | 34.7 | 16.6 | 41.5 | 10.5 | 6.9 | 16.2 |
| Benin | 2011 | 29.0 | 26.9 | 28.5 | 12.6 | 7.5 | 18.6 |
| Benin | 2014 | 25.0 | 15.8 | 29.7 | 20.4 | 18.6 | 24.0 |
| Benin | 2017 | 28.2 | 26.6 | 28.6 | 18.1 | 18.1 | 19.4 |
| Burkina Faso | 2003 | 60.8 | 34.1 | 70.7 | 19.8 | 8.0 | 39.1 |
| Burkina Faso | 2010 | 61.3 | 39.2 | 68.5 | 34.2 | 20.0 | 49.8 |
| Burundi | 2010 |  |  |  | 33.6 | 34.3 | 36.9 |
| Burundi | 2016 |  |  |  | 49.4 | 54.2 | 49.1 |
| Cameroon | 2004 | 55.8 | 41.6 | 66.1 | 32.1 | 9.8 | 49.4 |
| Cameroon | 2011 | 59.5 | 48.6 | 69.1 | 32.7 | 12.0 | 43.7 |
| Cameroon | 2014 | 77.8 | 66.6 | 84.4 | 49.1 | 42.0 | 54.0 |
| Chad | 2004 | 29.4 |  |  | 5.0 | 20.0 | 11.9 |
| Chad | 2010 | 24.0 |  |  | 13.8 | 10.3 | 12.5 |
| Chad | 2014 | 20.8 |  |  | 10.8 |  | 15.0 |
| Congo | 2005 | 27.0 | 28.2 | 32.7 | 19.5 | 15.0 | 27.8 |
| Congo | 2011 | 50.7 | 33.0 | 62.1 | 34.4 | 27.9 | 44.5 |
| Congo | 2014 | 41.7 | 40.2 | 39.7 | 33.0 | 28.4 | 37.2 |
| Congo DR | 2007 | 27.6 | 26.6 | 26.2 | 10.4 | 5.4 | 18.8 |
| Congo DR | 2010 | 18.1 | 5.1 | 26.6 | 15.3 | 16.1 | 18.3 |
| Congo DR | 2013 | 24.2 | 18.8 | 26.5 | 15.8 | 9.9 | 24.5 |
| Cote d’Ivoire | 2011 | 33.6 | 24.6 | 39.0 | 22.2 | 19.4 | 29.5 |
| Cote d’Ivoire | 2016 | 37.4 | 24.6 | 46.6 | 24.5 | 18.2 | 32.8 |
| Eswatini | 2006 | 62.6 | 44.0 | 70.7 | 59.5 | 55.2 | 65.3 |
| Eswatini | 2014 | 85.9 | 83.7 | 91.9 | 77.9 | 80.6 | 84.8 |
| Ethiopia | 2000 | 55.0 |  |  | 10.5 | 3.4 | 21.1 |
| Ethiopia | 2005 |  |  |  | 26.0 | 12.1 | 41.2 |
| Ethiopia | 2011 | 56.2 |  |  | 53.0 | 35.4 | 69.1 |
| Ethiopia | 2016 | 65.6 |  |  | 65.6 | 46.8 | 81.0 |
| Gabon | 2000 | 34.1 | 21.5 | 40.4 | 20.6 | 15.7 | 23.9 |
| Gabon | 2012 | 57.3 | 49.9 | 65.5 | 33.3 | 27.9 | 31.5 |
| Gambia | 2010 |  |  |  | 19.8 | 15.0 | 22.1 |
| Gambia | 2013 |  |  |  | 17.1 | 11.8 | 25.2 |
| Ghana | 2003 | 36.8 |  | 43.5 | 22.9 | 16.5 | 34.8 |
| Ghana | 2008 | 31.0 | 27.9 | 30.7 | 23.5 | 19.0 | 26.8 |
| Ghana | 2011 | 47.0 | 39.0 | 56.9 | 38.2 | 34.9 | 49.3 |
| Ghana | 2014 | 36.1 | 41.9 | 36.8 | 36.6 | 40.4 | 35.2 |
| Guinea | 2005 | 42.1 |  |  | 18.6 | 13.5 | 28.1 |
| Guinea | 2012 | 39.3 |  |  | 11.4 | 15.2 | 11.1 |
| Guinea | 2016 | 33.7 |  |  | 20.2 | 16.1 | 21.3 |
| Guinea | 2018 | 56.8 |  |  | 35.2 | 23.5 | 44.1 |
| Kenya | 2003 | 45.6 |  |  | 33.8 | 20.4 | 45.0 |
| Kenya | 2009 | 48.0 |  |  | 44.8 | 29.4 | 59.4 |
| Kenya | 2014 | 63.4 |  |  | 66.5 | 55.6 | 76.1 |
| Lesotho | 2004 | 58.9 |  |  | 45.3 | 31.9 | 58.5 |
| Lesotho | 2009 | 55.0 |  |  | 57.0 | 48.1 | 65.8 |
| Lesotho | 2014 | 70.6 |  |  | 68.4 | 63.9 | 74.3 |
| Liberia | 2007 | 26.0 | 7.6 | 32.9 | 11.0 | 5.7 | 13.3 |
| Liberia | 2013 | 38.4 | 30.4 | 38.2 | 32.9 | 20.2 | 44.4 |
| Madagascar | 2003 | 32.5 |  |  | 36.8 | 21.7 | 48.5 |
| Madagascar | 2008 | 27.9 |  |  | 43.6 | 37.4 | 46.3 |
| Malawi | 2000 | 28.7 | 27.8 | 36.3 | 36.8 | 32.2 | 45.4 |
| Malawi | 2004 | 31.8 | 30.0 | 40.1 | 40.6 | 34.5 | 48.0 |
| Malawi | 2010 | 45.1 | 31.0 | 59.0 | 54.1 | 53.3 | 57.8 |
| Malawi | 2013 | 40.8 | 39.0 | 44.6 | 72.0 | 70.4 | 74.3 |
| Malawi | 2015 | 43.8 | 38.3 | 45.2 | 71.5 | 70.8 | 73.1 |
| Mali | 2001 | 26.0 |  |  | 15.1 | 8.1 | 25.3 |
| Mali | 2006 | 36.9 |  |  | 17.6 | 8.0 | 25.9 |
| Mali | 2012 | 34.5 |  |  | 26.6 | 16.7 | 37.8 |
| Mali | 2015 | 40.9 |  |  | 28.1 | 9.6 | 43.8 |
| Mali | 2018 | 43.4 |  |  | 38.8 | 26.0 | 53.0 |
| Mozambique | 2003 | 59.1 | 43.2 | 66.9 | 49.7 | 48.1 | 50.4 |
| Mozambique | 2011 | 39.8 | 3.7 | 54.8 | 28.6 | 17.5 | 39.4 |
| Mozambique | 2015 | 67.5 |  | 73.8 | 44.6 | 33.1 | 55.8 |
| Namibia | 2000 | 65.2 | 54.7 | 76.9 | 59.0 | 53.1 | 65.6 |
| Namibia | 2006 | 82.5 | 69.9 | 91.5 | 65.0 | 54.9 | 75.2 |
| Namibia | 2013 | 79.9 | 75.2 | 82.3 | 66.1 | 58.9 | 72.5 |
| Niger | 2006 |  |  |  | 15.2 | 8.0 | 23.8 |
| Niger | 2012 |  |  |  | 35.9 | 31.4 | 44.0 |
| Nigeria | 2003 | 42.6 | 32.5 | 57.0 | 23.4 | 13.2 | 36.1 |
| Nigeria | 2008 | 46.3 | 25.8 | 55.1 | 19.9 | 10.6 | 30.6 |
| Nigeria | 2013 | 61.3 | 31.2 | 70.5 | 20.1 | 5.5 | 35.0 |
| Nigeria | 2016 | 32.7 | 17.6 | 42.9 | 18.0 | 11.0 | 32.1 |
| Nigeria | 2018 | 28.4 | 20.9 | 31.6 | 27.4 | 17.0 | 34.6 |
| Rwanda | 2000 | 35.9 |  |  | 11.4 | 6.1 | 16.6 |
| Rwanda | 2005 | 25.2 |  |  | 15.8 | 9.9 | 25.0 |
| Rwanda | 2010 |  |  |  | 69.1 | 59.9 | 73.3 |
| Rwanda | 2014 |  |  |  | 72.0 | 74.8 | 68.5 |
| Senegal | 2005 |  |  |  | 15.8 | 8.0 | 24.8 |
| Senegal | 2010 | 27.2 |  |  | 19.1 | 9.5 | 28.2 |
| Senegal | 2012 |  |  |  | 21.3 | 10.8 | 34.2 |
| Senegal | 2014 | 42.9 |  |  | 26.6 | 15.4 | 40.7 |
| Senegal | 2015 |  |  |  | 34.8 | 16.9 | 52.7 |
| Senegal | 2016 |  |  |  | 35.0 | 26.6 | 51.0 |
| Senegal | 2017 |  |  |  | 37.1 | 26.6 | 55.8 |
| Sierra Leone | 2008 | 32.6 |  | 37.1 | 12.0 | 6.3 | 18.0 |
| Sierra Leone | 2013 | 63.9 | 55.4 | 69.2 | 29.8 | 26.9 | 39.4 |
| Sierra Leone | 2017 | 65.0 | 52.0 | 70.7 | 38.0 | 27.4 | 50.1 |
| Tanzania | 2005 | 44.5 | 28.1 | 54.6 | 34.9 | 25.4 | 50.2 |
| Tanzania | 2010 | 50.5 | 43.7 | 54.4 | 43.1 | 32.3 | 54.7 |
| Tanzania | 2015 | 51.9 | 41.0 | 51.4 | 47.7 | 41.7 | 54.8 |
| Togo | 2010 | 43.3 | 23.9 | 48.2 | 20.3 | 16.9 | 30.9 |
| Togo | 2013 | 44.3 | 40.5 | 50.5 | 24.8 | 13.7 | 31.1 |
| Uganda | 2000 | 63.3 |  |  | 29.0 | 17.6 | 46.1 |
| Uganda | 2006 | 52.5 |  |  | 26.1 | 17.6 | 42.0 |
| Uganda | 2011 | 47.9 |  |  | 33.6 | 24.5 | 45.3 |
| Uganda | 2016 | 53.6 |  |  | 46.9 | 35.5 | 60.1 |
| Zambia | 2001 | 35.8 | 16.7 | 50.4 | 38.7 | 21.8 | 53.9 |
| Zambia | 2007 | 48.1 | 42.3 | 56.8 | 49.6 | 45.4 | 61.5 |
| Zambia | 2014 | 34.7 | 33.8 | 42.1 | 62.4 | 53.0 | 74.9 |
| Zimbabwe | 2005 | 64.4 |  |  | 76.9 | 65.8 | 80.1 |
| Zimbabwe | 2010 | 62.8 |  |  | 76.1 | 73.2 | 80.5 |
| Zimbabwe | 2014 | 62.8 |  |  | 85.7 | 81.7 | 86.4 |
| Zimbabwe | 2015 | 71.6 |  |  | 83.7 | 85.2 | 84.9 |

Based on the most recent surveys from 2000; AGYW – Adolescent Girls and Young Women aged 15-24 years; DFPSm – Demand for family planning satisfied by modern contraceptive methods; Poorest – The lowest tertile obtained from assets-ownership wealth-related index from principal component analysis. Richest – highest tertile obtained from assets-ownership wealth-related index from principal component analysisSub-category with sample size less than 30 observation excluded from analysis.
