# Supplementary material for: Wealth-related inequalities in demand for family planning satisfied among married and unmarried adolescent girls and young women in sub-Saharan Africa
Source: Reprod Health. 2021 Jun 17;18(Suppl 1):116. doi: 10.1186/s12978-021-01076-0 (PMC8210345; doi:10.1186/s12978-021-01076-0)
Supplement: Supplementary file 4 — Additional file 4: Table S1. Data sources and and sample sizes by year of survey and marital status. [file 12978_2021_1076_MOESM4_ESM.docx]

Figure S2: Annual average rate of change of DFPSm among married AGYW (overall, poorest and richest) by country.

| 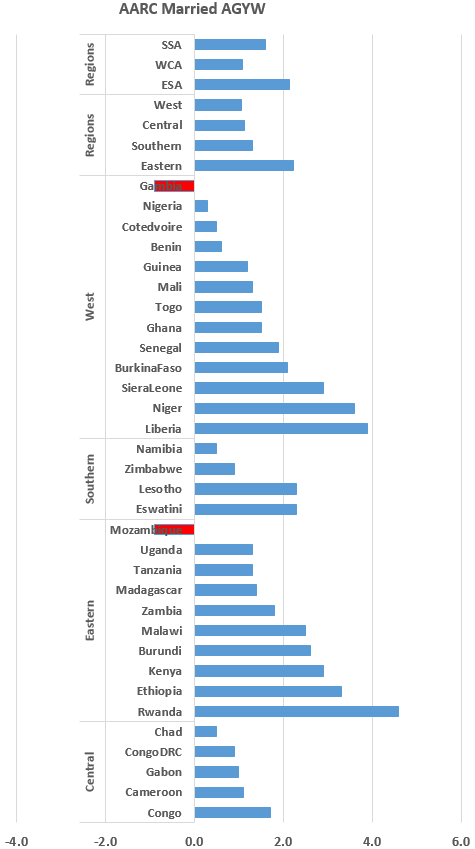 | 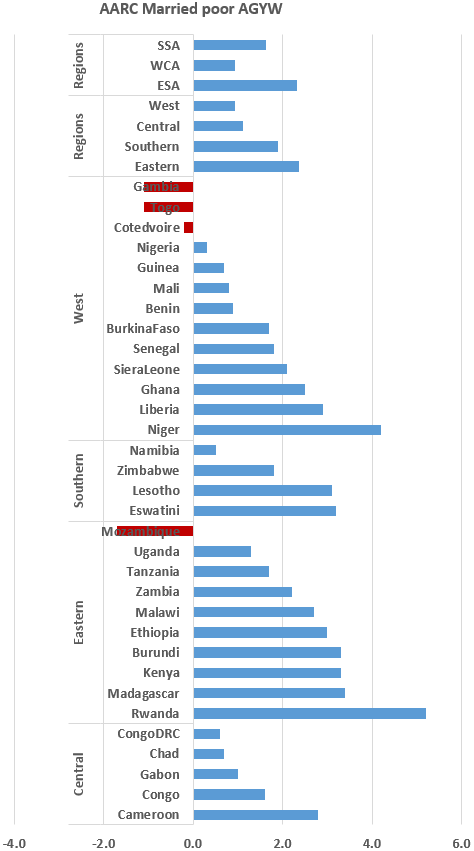 | 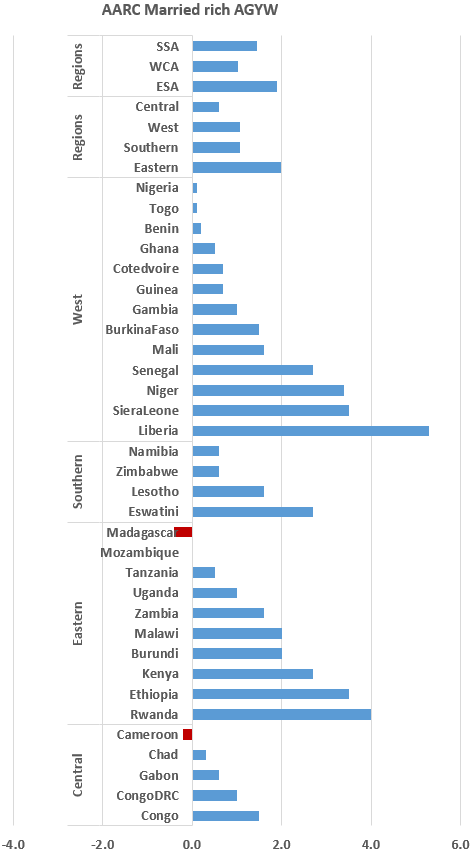 |
| --- | --- | --- |

Based on the most recent surveys from 2000; AGYW – Adolescent Girls and Young Women aged 15-24 years; DFPSm – Demand for family planning satisfied by modern contraceptive methods; Poorest – The lowest tertile obtained from assets-ownership wealth-related index from principal component analysis. Richest – highest tertile obtained from assets-ownership wealth-related index from principal component analysis; AAARC- Annual average rate of change of DFPSm. Sub-category with sample size less than 30 observation excluded from analysis.
